# Entwicklung der ambulanten psychotherapeutischen Versorgung in Sachsen

**DOI:** 10.1007/s00115-025-01805-x

**Published:** 2025-03-05

**Authors:** Lilly Rüthrich, Tina Haase, Lorenz Harst, Markus Kösters

**Affiliations:** Zentrum für Evidenzbasierte Gesundheitsversorgung (ZEGV) – Zweigstelle am Medizincampus Chemnitz der TU Dresden, Universitätsklinikum und Medizinische Fakultät Carl Gustav Carus, Technische Universität Dresden, Dresden, Deutschland

**Keywords:** Therapeut:innenquote, Psychotherapie, Auslastung, Kassenzulassung, Erreichbarkeit, Therapist quota, Psychotherapy, Utilization, Health insurance approval, Accessibility

## Abstract

**Hintergrund:**

Die Versorgung psychisch erkrankter Menschen sollte bedarfsgerecht, zeit- und wohnortnah erfolgen. Diesem Anspruch muss insbesondere die ambulante Versorgungsstruktur gerecht werden. Vor diesem Hintergrund wird in dieser Untersuchung die Entwicklung des kassenärztlichen psychotherapeutischen Versorgungsangebotes in Sachsen dargestellt.

**Methodik:**

Die ambulanten Zulassungsdaten für die fachärztlichen und psychotherapeutischen Fachgruppen aus dem Arztregister der Kassenärztlichen Vereinigung Sachsen wurden retrospektiv im Zeitraum 2014 bis 2023 systematisch aufbereitet, deskriptiv und mittels Geodatenanalyse ausgewertet. Anhand der öffentlich verfügbaren Daten der Geodatenbank Sachsen wurden Entfernungsanalysen zwischen allen sächsischen Ortsteilen und den Niederlassungen durchgeführt.

**Ergebnisse:**

Die Anzahl der niedergelassenen Psychotherapeut:innen ist von 2014 bis 2023 deutlich gestiegen, insbesondere auch in den ländlichen Regionen. So wurde im Erwachsenenbereich ein Anstieg der Anzahl an niedergelassenen Psychotherapeut:innen von 761 auf 1224 verzeichnet. Im Jahr 2023 war für alle Ortsteile in Sachsen eine Erreichbarkeit psychotherapeutischer Praxen im Erwachsenenbereich innerhalb von 20 km gegeben, sodass sich das Versorgungsangebot hinsichtlich der räumlichen Abdeckung verbesserte. Das Versorgungsangebot der Kinder- und Jugendlichen-Psychotherapeut:innen (KJP) und Fachärzt:innen im KJP- und Erwachsenenbereich verbesserte sich hingegen nur geringfügig.

**Diskussion:**

Das Versorgungsangebot hat sich im städtischen und ländlichen Raum verbessert. Jedoch fehlen Daten, um die Bedarfsdeckung belastbar zu prüfen. Eine Analyse des psychotherapeutischen Behandlungsbedarfes ist daher dringend geboten.

**Zusatzmaterial online:**

Zusätzliche Informationen sind in der Online-Version dieses Artikels (10.1007/s00115-025-01805-x) enthalten.

## Abstract (en)

**Background:**

The care of mentally ill people should be carried out in a timely, needs-based and community-based manner. The structure of the outpatient care is a cornerstone in achieving this goal. Against this background, this study presents the development of psychotherapeutic care in Saxony.

**Methods:**

Data on outpatient psychotherapy between 2014 and 2023 from the medical register of the Saxony Association of Statutory Health Insurance Physicians were retrospectively evaluated and mapped using geodata analyses. Publicly available data from the Saxony geodatabase were used to analyze distances between local districts in Saxony and the private practices.

**Results:**

The number of psychotherapists in private practice increased between 2014 and 2023, particularly in rural regions. The number of psychotherapists for adults increased from 761 to 1224. In 2023 all psychotherapeutic practices for adults were within 20 km of all districts in Saxony so that the provision of care improved with respect to the spatial coverage. In contrast, the availability of pediatric and adolescent psychotherapists and specialists in the child and adolescent sector as well as for adults has improved only slightly.

**Discussion:**

The number of practices for psychotherapy has improved in urban and rural areas; however, there is a lack of data to reliably assess the coverage of needs. An analysis of the need for psychotherapeutic treatment is therefore urgently required.

Berichte über lange Wartezeiten, schlechte ländliche Versorgung und die Altersstruktur der Behandelnden lassen den Eindruck entstehen, dass sich das psychotherapeutische Versorgungsangebot in den letzten Jahren verschlechtert hat. Die ambulante Versorgungsstruktur sollte jedoch dem Anspruch gerecht werden, psychisch erkrankte Menschen bedarfsgerecht, zeit- und wohnortnah zu versorgen. Vor diesem Hintergrund wird in dieser Untersuchung die Entwicklung des kassenärztlichen psychotherapeutischen Versorgungsangebotes in Sachsen zwischen 2014 und 2023 dargestellt.

## Hintergrund und Fragestellung

Gemäß der Forderung der Bundesregierung benötigen Menschen mit psychischen Erkrankungen eine zeitnahe, wohnortnahe und bedarfsgerechte Behandlung [5]. Gleichzeitig geht die ambulante Versorgung mit einer dynamischen Entwicklung des Versorgungsangebotes kassenärztlicher (Kinder- und Jugendlichen‑)Psychotherapeut:innen und Fachärzt:innen einher. Die Zulassungsmöglichkeiten der Praxissitze je Planungsbereich bzw. Außenstellen, in welcher die hauptsächliche Tätigkeit erbracht wird, werden dabei seitens der Kassenärztlichen Bundesvereinigung sowie der zuständigen Kassenärztlichen Vereinigung je Bundesland anhand der Versorgungsgrade in der Bedarfsplanung festgelegt [9]. Dabei werden neben der Berechnung der Summe der Tätigkeitsumfänge im Planungsbereich unter anderem das Verhältnis der Einwohner:innen auf eine:n Fachärztin/-arzt, die regionale Morbidität (morbiditätsorientierter Risikostrukturausgleich), infrastrukturelle Besonderheiten sowie demografische, sozioökonomische und räumliche Faktoren einbezogen [2, 8, 9].

Zuletzt berichteten Studien, dass der psychotherapeutische Therapiebedarf unter anderem durch die COVID-19-Pandemie gestiegen ist und sich die Wartezeiten auf eine psychotherapeutische Therapie verlängert haben [13, 18]. Diese Wartezeiten könnten zu einer Verschlimmerung oder Chronifizierung psychischer Erkrankungen beitragen [4]. Eine Auswertung von Patient:innendaten der gesetzlichen Krankenversicherungen aus Bayern ergab eine durchschnittliche Wartezeit vom Erstkontakt bis zum Therapiebeginn von rund 14 Wochen [14]. In Sachsen betrug die Wartezeit auf ein ambulantes psychotherapeutisches Erstgespräch im Jahr 2011 durchschnittlich 14 Wochen, obwohl diese gemäß der Leitlinien 3 Wochen nicht überschreiten sollte [4]. Dabei zeigen sich u. a. in Sachsen insbesondere bei Kinder- und Jugendlichenpsychotherapeut:innen [18] und in ländliche Regionen [4] lange Wartezeiten. Im Rahmen des Gesetzes zur Stärkung der Versorgung in der gesetzlichen Krankenversicherung (GKV-Versorgungsstärkungsgesetz – GKV-VSG) vom 16.07.2015 wurde daher die Kassenärztliche Vereinigung zur Prüfung von Versorgungsaufträgen beauftragt [3]. Das laut § 19a der Zulassungsverordnung für Vertragsärzt:innen geforderte Zeitbudget beträgt 25 h Sprechstundenzeiten pro Woche bzw. laut des Praxispanels des Zentralinstituts der Kassenärztlichen Versorgung [19] 36,5 h Wochenarbeitszeit. Gleichzeitig wurde seitens der Kassenärztlichen Vereinigung Sachsen ein starker Anstieg der Nachfrage sowohl nach einem psychotherapeutischen Versorgungsauftrag als auch nach psychotherapeutischen Leistungen wahrgenommen [10, 18], sodass seit 2017 freie psychotherapeutische Versorgungsaufträge lediglich mit hälftigem Tätigkeitsumfang ausgeschrieben werden und demnach der Anteil an vollen Versorgungsaufträgen kontinuierlich sinkt.

Angesichts der dynamischen Entwicklung der Behandlungsangebote soll in dieser Studie das aktuelle Versorgungsangebot und die Entwicklung der Erreichbarkeit kassenärztlicher psychotherapeutischer Fachgruppen für das Bundesland Sachsen untersucht werden. Dabei sollen im Speziellen folgende Fragen beantwortet werden:

1. Wie hat sich die Behandlungskapazität, gemessen an der Anzahl der Niederlassungen und der aggregierten Tätigkeitsumfänge, in Sachsen zwischen 2014 und 2023 entwickelt?
2. Gab es im Untersuchungszeitraum regionale Unterschiede in der psychotherapeutischen Versorgung in Sachsen (Unterschiede zwischen Landkreisen sowie zwischen städtischem und ländlichem Raum)?
3. Bestanden Unterschiede im Versorgungsangebot für Erwachsene und für Kinder und Jugendliche, bezogen auf Niederlassungen und Tätigkeitsumfänge sowie auf regionale Verortung?

Die Ergebnisse sollen als Grundlage dienen, um zu prüfen, ob sich darauf aufbauend Hinweise auf die zu erwartende Entwicklung einer adäquaten, wohnortnahen psychotherapeutischen Versorgung im ambulanten Setting in Sachsen ableiten lassen.

## Studiendesign und Untersuchungsmethoden

Um das aktuelle psychotherapeutische Versorgungsangebot in Sachsen adäquat abzubilden, wurden die ambulanten Zulassungs- und Bedarfsplanungsdaten

- zum Fachgebiet,
- zur Tätigkeitsart,
- zum Planungsbereich/-adresse,
- zu den bedarfsplanungsrelevanten Tätigkeitsumfängen und deren Summe im Planungsbereich,
- zum relevanten Versorgungsgrad im Planungsbereich,
- zu den Zulassungsmöglichkeiten/-grenzen,
- zur Auslastung,
- zu den soziodemografischen Daten und
- zu den Zusatzbezeichnungen der Bedarfsplanungsarztgruppen

bei der Kassenärztlichen Vereinigung Sachsen beantragt. Das methodische Vorgehen folgt der Gute-Praxis-Sekundärdatenanalyse nach Swart et al. [17]. Ergänzend zu den Zulassungsdaten der Kassenärztlichen Vereinigung Sachsen wurden die Kontaktdaten und Informationen der Fachkunde der psychotherapeutischen Privatpraxen in Sachsen anhand der öffentlich zugänglichen Listen der Ostdeutschen Psychotherapeutenkammer [12] des Jahres 2023 ausgewertet, um zusätzlich das privatärztliche Versorgungsangebot abzubilden.

### Anzahl der Niederlassungen und aggregierten Tätigkeitsumfänge

Die Daten für die kassenärztlichen Fachgruppen in Sachsen wurden, aufgrund der unterschiedlichen Behandlungskapazitäten, differenziert nach psychotherapeutischen und fachärztlichen Fachpersonen aufbereitet, analysiert und interpretiert. Anhand der verfügbaren Daten aus dem Arztregister wurde anschließend die Entwicklung der Anzahl der Niederlassungen analysiert. Es wurde sowohl die absolute Anzahl an Niederlassungen (unabhängig vom Tätigkeitsumfang) als auch der aggregierte Tätigkeitsumfang ausgewertet. Der aggregierte Tätigkeitsumfang entspricht der Summe der Anteile der Versorgungsaufträge und damit der rechnerischen Behandlungskapazität. Die Datenaufbereitung und deskriptive Auswertung der ambulanten Zulassungsdaten fand dabei unter Verwendung von Tableau [15] retrospektiv für den Zeitraum von 2014 bis 2023 auf Landkreisebene statt, um einen kontinuierlichen Vergleich zu ermöglichen.

### Regionale Unterschiede

Da eine Analyse auf Ebene der Landkreise oder Postleitzahlgebiete aufgrund der geringen Auflösung des Raumbezuges für eine Entfernungsanalyse nicht aufschlussreich erschien, wurden zusätzlich die Jahre 2014 und 2023 mittels einer Geodatenanalyse in Tableau auf Ortsteilebene verglichen. Dabei wurden aus der Geodatenbank des Freistaates Sachsen die Zentroide (geographische Mittelpunkte) der insgesamt 3631 sächsischen Ortsteile gebildet, sodass eine detaillierte Analyse möglich ist. Ein Ortsteil ist „ein abgegrenzter und mit eigenem Namen versehener Teil der Stadt oder Gemeinde […]“ [11]. Anhand der Zentroide der sächsischen Ortsteile wurden Entfernungsanalysen durchgeführt, welche die Erreichbarkeit der im Umkreis von 20 km liegenden Niederlassungen der jeweiligen Fachgruppen einschließen. Zweigstellen wurden entsprechend der zugrunde liegenden Daten der KV Sachsen berücksichtigt. Daraufhin wurden Karten für die jeweiligen Fachgruppen für die Jahre 2014 und 2023 erstellt, welche die Ortsteile, die im definierten Umkreis keine Praxis enthalten, abbilden.

### Unterschiede in der Versorgung Erwachsener und von Kindern und Jugendlichen

Es wurden die in Sachsen niedergelassenen Fachärzt:innen und Psychotherapeut:innen in die Stichprobe als Studienpopulation eingeschlossen, differenziert nach dem Erwachsenen- sowie Kinder- und Jugendlichenbereich (nachfolgend KJP). Dabei wurden die Gruppen anhand ausdifferenzierter Fachgebietsbezeichnungen gebildet. In die Gruppe der Psychotherapeut:innen im Erwachsenenbereich wurden die psychologischen Psychotherapeut:innen und psychotherapeutisch tätigen Ärzt:innen zusammengefasst. Die Kinder- und Jugendlichenpsychotherapeut:innen wurden aus den ausschließlich im KJP-Bereich tätigen Psychotherapeut:innen und psychotherapeutisch tätigen Ärzt:innen gebildet. Die Gruppe der Fachärzt:innen im Erwachsenenbereich umfasst die Fachgebiete der Nervenheilkunde, Neurologie, Psychiatrie, Psychotherapie, psychosomatischen Medizin und psychotherapeutischen Medizin. Im fachärztlichen KJP-Bereich wurden die Fachärzt:innen der Kinder‑/Jugendpsychiatrie und -psychotherapie eingruppiert.

## Ergebnisse

### Die Anzahl der Niederlassungen und summierten Tätigkeitsumfänge stiegen zwischen 2014 und 2023 sowohl im Erwachsenen- als auch im KJP-Bereich

Zum Stichtag 01.01.2023 war mit 75,5 % der Großteil der insgesamt 2161 eingeschlossenen kassenärztlichen Psychotherapeut:innen und Fachärzt:innen in einer Einzelpraxis tätig. 8,4 % waren an einem Medizinischen Versorgungszentrum (MVZ) tätig. In Anstellung befanden sich 7,9 % der Ärzt:innen/Psychotherapeut:innen. 3,8 % arbeiteten in einer Gemeinschaftspraxis und 1,7 % waren (instituts-)ermächtigt. 0,9 % waren an einem Krankenhaus oder einer sonstigen Einrichtung angestellt, was die Vertragsärzt:innen in Psychiatrischen Institutsambulanzen (PIA) umfasst, die erstmalig zum Stichtag 01.10.2014 angerechnet wurden. In eTabelle 1 der Anlage im Zusatzmaterial online wird die Stichprobe zusätzlich anhand der Zusatzqualifikationen der Psychotherapeut:innen und Fachärzt:innen beschrieben.

**Abb. 1:**
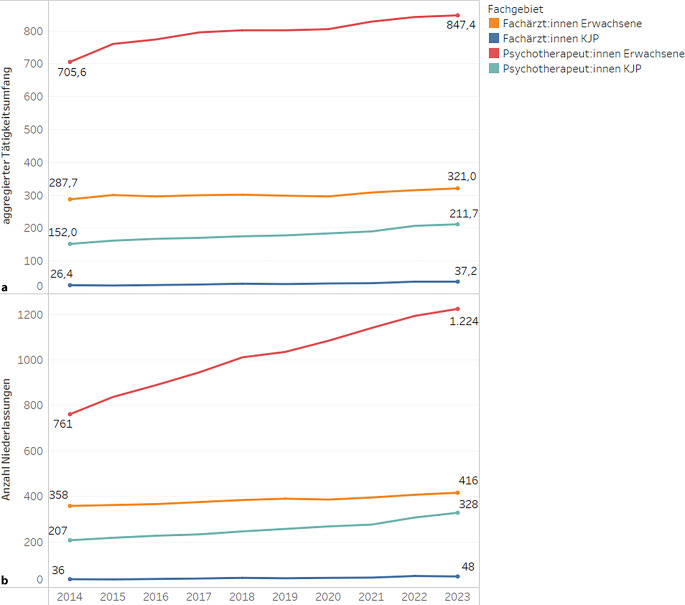
Entwicklung der Anzahl an Niederlassungen (**a**) und des aggregierten Tätigkeitsumfangs (**b**) von 2014 bis 2023 (Zulassungsdaten der Kassenärztlichen Vereinigung Sachsen). *KJP*: Kinder- und Jugendlichenbereich

Die Analyse der Zulassungsdaten des ambulanten psychotherapeutischen Versorgungsangebotes in Sachsen zeigte von 2014 bis 2023 einen deutlichen Anstieg der Anzahl an niedergelassenen Psychotherapeut:innen und Fachärzt:innen. So stieg die Anzahl an niedergelassenen Psychotherapeut:innen im Erwachsenenbereich von 761 im Jahr 2014 auf 1224 Psychotherapeut:innen im Jahr 2023. In der KJP war im gleichen Zeitraum ein Anstieg von 207 auf 328 Psychotherapeut:innen zu verzeichnen. Relativ gesehen stieg die Anzahl der niedergelassenen Psychotherapeut:innen für Erwachsenen- und für die KJP vergleichbar (+61 % bzw. +58 % Niederlassungen, vgl. eTabelle 2 im Zusatzmaterial online) an. Vergleichend dazu stieg im gleichen Zeitraum die Anzahl an Fachärzt:innen für den Erwachsenenbereich von 358 auf 416 und an Fachärzt:innen für KJP von 36 auf 48 an. Die Anzahl an niedergelassenen Fachärzt:innen stieg hingegen im Erwachsenenbereich geringer an als im KJP-Bereich an (+16 % bzw. +33 Niederlassungen). In Abb. 1 wird die Entwicklung der Anzahl an Niederlassungen und des Tätigkeitsumfangs von 2014 bis 2023 dargestellt.

Im gleichen Zeitraum stieg auch der aggregierte Tätigkeitsumfang (Summe der Anteile an einem Versorgungsauftrag) der jeweiligen Fachgruppen, jedoch im geringeren Ausmaß. So gab es einen Anstieg des gesamten Tätigkeitsumfangs der Psychotherapeut:innen im Erwachsenenbereich von 706 auf 847 und in der KJP von 152 auf 212. Die Summe der Tätigkeitsumfänge der Fachärzt:innen für Erwachsene stieg von 288 auf 321, die Tätigkeitsumfänge der Fachärzt:innen für KJP stieg von 26 auf 37. Dies entspricht einem geringeren Anstieg der aggregierten Tätigkeitsumfänge im Erwachsenen- im Vergleich zum KJP-Bereich, sowohl für die Psychotherapeut:innen (+20 % bzw. +39 %) als auch für die Ärzt:innen (+11 % bzw. +42 %). Mit Ausnahme der Ärzt:innen im KJP-Bereich waren die Anstiege der aggregierten Tätigkeitsumfänge deutlich geringer als die Anstiege der Niederlassungen. Darüber hinaus ist festzustellen, dass sich der Anteil der Niederlassungen mit vollem Tätigkeitsumfang für den Bereich der Psychotherapeut:innen im Auswertungszeitraum deutlich reduziert hat, sodass im Jahr 2023 etwa 50 % der Psychotherapeut:innen einen hälftigen (oder geringeren) Versorgungsauftrag ausübten (vgl. eAbbildung 1 der Anlage im Zusatzmaterial online).

Ergänzend zu den aufgezeigten Ergebnissen wurden anhand der Auflistung der Ostdeutschen Psychotherapeutenkammer [12] in Sachsen 102 Privatpraxen im psychotherapeutischen Erwachsenenbereich (Stand 05.10.2023) und 12 Privatpraxen der KJP (Stand 03.05.2023) identifiziert. Da keine retrospektiven Daten zur Verfügung stehen, wurden die Privatpraxen bei der Datenanalyse nicht berücksichtigt.

### Das Versorgungsangebot hat sich sowohl im städtischen als auch im ländlichen Raum zwischen 2017 und 2023 verbessert

In Bezug auf die Entwicklung des psychotherapeutischen Versorgungsangebotes innerhalb der sächsischen Landkreise ist seit 2014 ein höheres psychotherapeutisches Angebot auch außerhalb der städtischen Ballungszentren erkennbar (siehe eTabelle 2 der Anlage im Zusatzmaterial online). Im Allgemeinen stieg im Zeitraum von 2014 bis 2023 sowohl die Anzahl an Psychotherapeut:innen und Fachärzt:innen im Erwachsenen- und KJP-Bereich als auch die Summe der Tätigkeitsumfänge pro Fachgebiet an. Lediglich in den Landkreisen Görlitz, Vogtlandkreis und Zwickau gab es in einzelnen Bereichen keinen Anstieg. Eine ausführliche Aufstellung der einzelnen Landkreise befindet sich in eTabelle 2 der Anlage im Zusatzmaterial online.

**Abb. 2:**
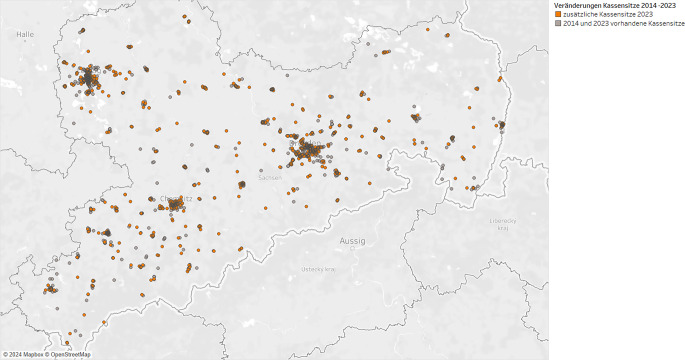
Entwicklung der psychotherapeutischen Niederlassungen von 2014 bis 2023 in Sachsen. (Zulassungsdaten der Kassenärztlichen Vereinigung Sachsen)

Die zeitliche Entwicklung der Erreichbarkeit kassenärztlicher Sitze im psychotherapeutischen Bereich ist in Abb. 2 dargestellt, wobei aufgegebene Niederlassungen nicht abgebildet werden. Es zeigt sich, dass insbesondere in den ländlichen Regionen mehr Niederlassungen zusätzlich entstanden.

**Abb. 3:**
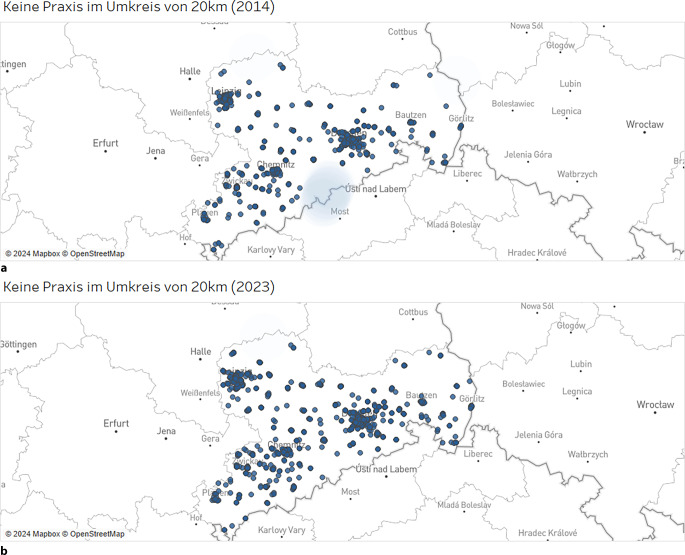
Entfernungsanalyse der niedergelassenen Psychotherapeut:innen im Erwachsenenbereich von 2014 (**a**) und 2023 (**b**). *Hellblau gefärbte* *Flächen* kennzeichnen Ortsteile ohne Praxis im Umkreis von 20 km. Blaue Punkte kennzeichnen die Niederlassungen. (Zulassungsdaten der Kassenärztlichen Vereinigung Sachsen/Geodatenbank des Freistaates Sachsen)

Die darauf aufbauenden Entfernungsanalysen ergaben, dass in allen Fachgruppen eine Verbesserung der räumlichen Verteilung des Versorgungsangebotes von 2014 bis 2023 erkennbar ist. So stieg der Anteil der sächsischen Ortsteile, die innerhalb eines Radius von 20 km eine:n niedergelassene:n Psychotherapeut:in im Erwachsenenbereich aufweisen, auf 100 % (2014: 3619/3631 und 2023: 3631/3631 Ortsteile mit Praxis im Umkreis von 20 km). Die Entwicklung ist beispielhaft in Abb. 3 erkennbar, in welcher die Ortsteile, die keine Praxis im benannten Umkreis haben, hellblau gefärbt sind. Gleichzeitig hat sich auch die Verteilung des psychotherapeutischen Versorgungsangebots in der KJP verbessert, sodass im Jahr 2023 lediglich ein sächsischer Ortsteil keine innerhalb von 20 km erreichbare psychotherapeutische Praxis für Kinder und Jugendliche aufwies (2014: 95/3631 und 2023: 1/3631 Ortsteile ohne Praxis im Umkreis von 20 km).

**Abb. 4:**
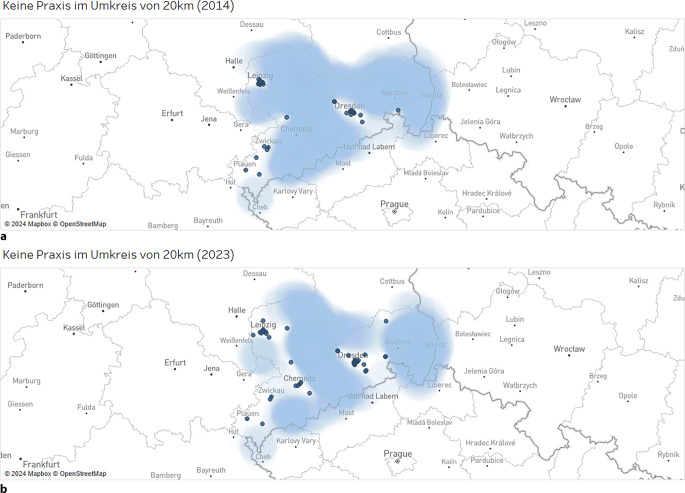
Entfernungsanalyse der niedergelassenen Fachärzt:innen im Kinder- und Jugendlichenbereich von 2014 (**a**) und 2023 (**b**). *Hellbau gefärbte Flächen* kennzeichnen Ortsteile ohne Praxis im Umkreis von 20 km. Blaue Punkte kennzeichnen die Niederlassungen. (Zulassungsdaten der Kassenärztlichen Vereinigung Sachsen/Geodatenbank des Freistaates Sachsen)

Die Fachärzt:innen der KJP waren lediglich in den Ballungsgebieten bzw. Großstädten gut vertreten. Die Anzahl der Ortsteile, die ein entsprechendes fachärztliches Versorgungsangebot im KJP-Bereich im Umkreis von 20 km aufwiesen, stieg zwar ebenfalls an, blieb aber insgesamt auf sehr niedrigem Niveau (2014: 2030/3631 und 2023: 2556/3631 Ortsteile mit Praxis im Umkreis von 20 km). Somit waren im Jahr 2023 noch 1075 von 3631 Ortsteilen ohne Praxis im Umkreis von 20 km (Abb. 4). Auch die Erreichbarkeit von Fachärzt:innen im Erwachsenenbereich verbesserte sich (2014: 16/3631 und 2023: 7/3631 Ortsteile ohne Praxis im Umkreis von 20 km).

## Diskussion

Diese Studie zeigt anhand der Zulassungsdaten der Kassenärztlichen Vereinigung Sachsen, der Ostdeutschen Psychotherapeutenkammern und anhand von Entfernungsanalysen, dass sich im Bundesland Sachsen das ambulante psychotherapeutische Versorgungsangebot zwischen 2014 und 2023 verbesserte: Sowohl im fachärztlichen als auch psychotherapeutischen Bereich verbesserte sich das Versorgungsangebot hinsichtlich der Anzahl der Niederlassungen und ihrer Erreichbarkeit. Dabei verbesserte sich das Versorgungsangebot flächendeckend, also sowohl in Städten als auch in ländlichen Regionen im Erwachsenen- aber auch im KJP-Bereich.

Die Reduktion der Tätigkeitsumfänge und die damit zu erwartende bessere Erfüllung der Versorgungsaufträge [1, 6] dürfte den in den Analysen aufgezeigten Anstieg der Behandlungskapazitäten hinsichtlich der real verfügbaren Therapieplätze weiter erhöht haben. Da die genaue Auslastung der Praxen jedoch für eine Analyse nicht zur Verfügung stand, kann der zusätzliche Anstieg der Behandlungskapazitäten nicht belastbar geschätzt werden. Gleichzeitig könnte sich das ambulante psychotherapeutische Versorgungsangebot aufgrund der prognostizierten Altersentwicklungen in den nächsten zwei Dekaden verschlechtern, da in dieser Studie seit dem Jahr 2014 ein Anstieg des Durchschnittsalters der in Sachsen tätigen ambulanten Psychotherapeut:innen und Fachärzt:innen verzeichnet wurde. Um die damit einhergehenden Folgen entsprechend bewerten und mögliche Gegenmaßnahmen ableiten zu können, sollten auf diese Untersuchung aufbauend Studien zur Niederlassungsbereitschaft einbezogen werden.

Da neue Niederlassungen sowie deren Kapazitäten und Auslastung nicht vollumfänglich bekannt sind, sollten die künftigen Entwicklungen im Sinne eines kontinuierlichen Versorgungsmonitorings weiterführend überprüft werden. Um zu beurteilen, ob die Behandlungskapazitäten künftig den notwendigen Bedarf an psychotherapeutischen Behandlungen decken können, ist sowohl eine zusätzliche Analyse der Entwicklung des psychotherapeutischen Behandlungsbedarfes als auch der stationären Versorgung, künftiger Prognosen und genauer Tätigkeitsfelder der jeweiligen Fachgruppen essenziell. Dadurch könnte die Gesamtversorgung beurteilt, kontinuierlich überwacht und regionale Maßnahmen der Versorgungssteuerung evidenzgeleitet entwickelt werden. Dabei sollte insbesondere untersucht werden, inwiefern sich Angebot und Nachfrage der Fachärzt:innen in der KJP entwickeln, da die hier größeren Entfernungen und demnach schlechteren Erreichbarkeiten der Praxen möglicherweise zu Engpässen führen könnten. Zudem sollte das gestufte Vorgehen (Stepped-care-Ansatz) aufgrund des Potenzials der evidenzbasierten, leitliniengerechten und netzwerkübergreifenden Versorgung bei der psychotherapeutischen Patient:innenbehandlung in die weiterführende Forschung einbezogen werden [7].

### Limitationen

In der vorliegenden Untersuchung wurden alle ambulanten, kassenärztlich tätigen Fachärzt:innen und Psychotherapeut:innen in Sachsen einbezogen. Das Geschlecht und die exakten Altersangaben der Praxisinhaber:innen konnten aus datenschutzrechtlichen Gründen nicht zur Verfügung gestellt werden, sodass Daten zum Alter nur in aggregierter Form vorlagen und somit keine Prognosen hinsichtlich der Altersentwicklung möglich waren. Ebenso waren keine Daten zur Auslastung der Praxen verfügbar, sodass nicht analysiert werden konnte, wie viele Patient:innen tatsächlich durch das Versorgungsangebot erreicht wurden. Anzumerken ist zudem, dass die zusammengefassten Fachärzt:innengruppen sehr heterogen in Bezug auf den Umfang der erbrachten psychotherapeutischen Leistungen sein dürften, für die Analysen jedoch keine Daten vorlagen, die eine Berücksichtigung dieser Unterschiede erlaubten. Ebenso sind Behandlungen, die z. B. im Rahmen einer Genehmigung zur Behandlung von Kindern und Jugendlichen erbracht wurden, nur dann erfasst, wenn sie mit einem Tätigkeitsumfang bei der KV erfasst sind. Aufgrund der fehlenden Datenbasis für die psychotherapeutisch tätigen Privatpraxen wurden die psychotherapeutischen Privatpraxen in weiterführenden Analysen nicht berücksichtigt. Darüber hinaus wurden die verfügbaren Daten im Rahmen der Entfernungsanalysen auf Ortsteilebene untersucht, sodass die sächsischen Bürger:innen nicht individuell berücksichtigt werden konnten. Der Umkreis von 20 km, welcher bei der räumlichen Entfernungsanalyse der Ortsteilzentroide und der Niederlassungen angewandt wurde, ist letztendlich arbiträr und berücksichtigt nicht die individuellen Unterschiede der Fortbewegungsmöglichkeit und individuelle Umsetzbarkeit der Patient:innen. Da im Rahmen von Entfernungsanalysen bisher keine Grenzen zur Festlegung von Umkreisen vorliegen, wurde sich an flexiblen Erreichbarkeitsmodellen orientiert, welche ebenfalls seitens der Regierungskommission genutzt werden [16]. Des Weiteren konnte bei an den Grenzen zu anderen Bundesländern liegenden Ortsteilen lediglich die Erreichbarkeit der sächsischen Niederlassungen berücksichtigt werden. Abschließend sollte in weiterführenden Analysen geprüft werden, inwiefern die dargebotenen Ergebnisse auf andere Studienpopulationen und Fachgebiete übertragen werden können.

## Fazit für die Praxis

- Das Versorgungsangebot hat sich im städtischen und ländlichen Raum grundsätzlich verbessert.
- Das Versorgungsangebot hat sich sowohl für Erwachsene als auch für Kinder und Jugendliche verbessert.
- Es fehlen Daten, um die Bedarfsdeckung belastbar zu prüfen. Eine Analyse des psychotherapeutischen Behandlungsbedarfes ist daher dringend geboten.

## Supplementary Information

Anlage

## Data Availability

Die Daten wurden von der Kassenärztlichen Vereinigung auf Antrag zur Verfügung gestellt und können dort nach Prüfung des berechtigten Interesses angefordert werden.
