# Supplementary material for: Development of outpatient psychotherapeutic care in Saxony
Source: Nervenarzt. 2025 Mar 5;96(7):669–77. [Article in German] doi: 10.1007/s00115-025-01805-x (PMC12662872; doi:10.1007/s00115-025-01805-x)

**Anlage**

Tabelle A1: Zusatzbezeichnungen der Psychotherapeut:innen und Fachärzt:innen
(Zulassungsdaten der Kassenärztlichen Vereinigung Sachsen)

| **Zusatzbezeichnung** | **Anzahl** | **Prozentzahl** |
| --- | --- | --- |
| Psychoanalyse | 203 | 15,5% |
| Akupunktur | 133 | 10,1% |
| Naturheilverfahren | 114 | 8,7% |
| Suchtmedizinische Grundversorgung | 101 | 7,7% |
| Chirotherapie | 96 | 7,3% |
| Rehabilitationswesen | 85 | 6,5% |
| Homöopathie | 72 | 5,5% |
| Spezielle Schmerztherapie | 70 | 5,3% |
| Geriatrie | 60 | 4,6% |
| Notfallmedizin | 45 | 3,4% |
| Klinische Neuropsychologie b. PT, K-J-PT | 40 | 3,1% |
| Physikalische Therapie | 34 | 2,6% |
| Intensivmedizin | 33 | 2,5% |
| Ärztliches Qualitätsmanagement | 31 | 2,4% |
| Palliativmedizin | 31 | 2,4% |
| Systemische Therapie bei Psychol., K-J-PT | 23 | 1,8% |
| Psychotherapie - fachgebunden | 22 | 1,7% |
| Sportmedizin | 21 | 1,6% |
| Manuelle Medizin/Chirotherapie | 15 | 1,1% |
| Allergologie | 10 | 0,8% |
| Betriebsmedizin | 10 | 0,8% |
| Medizinische Informatik | 10 | 0,8% |
| Psychodiabetologie bei Psychol., K-J-PT | 10 | 0,8% |
| Schlafmedizin | 10 | 0,8% |
| Umweltmedizin | 10 | 0,8% |
| Neuropsychologie bei Psychol., K-J-PT | 8 | 0,6% |
| Physikalische Therapie und Balneologie | 5 | 0,4% |
| Medikamentöse Tumortherapie | 4 | 0,3% |
| Spez.Schmerzpsychoth.bei Psychol., K-J-PT | 4 | 0,3% |
| Kinder-Orthopädie | 1 | 0,1% |
| Manuelle Medizin | 1 | 0,1% |
| Spezielle Orthopädische Chirurgie | 1 | 0,1% |

Tabelle A2: Entwicklung des psychotherapeutischen Versorgungsangebotes nach sächsischen Landkreisen 2014-2023 (Zulassungsdaten der Kassenärztlichen Vereinigung Sachsen)

|  |  | Anzahl Niederlassungen | | Veränderung (%) | |  | Aggregierter Tätigkeitsumfang | | Veränderung (%) |
| --- | --- | --- | --- | --- | --- | --- | --- | --- | --- |
|  | **Fachgebiet** | **2014** | **2023** |  |  | | **2014** | **2023** |  |
| Bautzen | Fachärzt:innen KJP |  | 4 |  |  | |  | 4,0 |  |
|  | Fachärzt:innen Erwachsene | 19 | 21 | 11% |  | | 15,0 | 17,5 | 17% |
|  | Psychotherapeut:innen Erwachsene | 39 | 71 | 82% |  | | 33,0 | 45,5 | 38% |
|  | Psychotherapeut:innen KJP | 7 | 18 | 157% |  | | 4,0 | 12,0 | 200% |
| Chemnitz | Fachärzt:innen KJP |  | 4 |  |  | |  | 3,5 |  |
|  | Fachärzt:innen Erwachsene | 28 | 34 | 21% |  | | 22,0 | 25,0 | 14% |
|  | Psychotherapeut:innen Erwachsene | 77 | 100 | 30% |  | | 71,5 | 77,5 | 8% |
|  | Psychotherapeut:innen KJP | 22 | 28 | 27% |  | | 16,5 | 17,5 | 6% |
| Dresden | Fachärzt:innen KJP | 12 | 13 | 8% |  | | 7,1 | 7,8 | 9% |
|  | Fachärzt:innen | 85 | 99 | 16% |  | | 70,6 | 72,0 | 2% |
|  | Psychotherapeut:innen Erwachsene | 185 | 290 | 57% |  | | 164,9 | 179,2 | 9% |
|  | Psychotherapeut:innen KJP | 51 | 68 | 33% |  | | 39,0 | 39,5 | 1% |
| Erzgebirgskreis | Fachärzt:innen KJP |  | 1 |  |  | |  | 1,0 |  |
|  | Fachärzt:innen Erwachsene | 17 | 19 | 12% |  | | 16,0 | 15,8 | -2% |
|  | Psychotherapeut:innen Erwachsene | 31 | 56 | 81% |  | | 30,5 | 44,5 | 46% |
|  | Psychotherapeut:innen KJP | 13 | 22 | 69% |  | | 9,0 | 17,5 | 94% |
| Görlitz | Fachärzt:innen KJP | 1 |  | -100% |  | | 0,5 |  | -100% |
|  | Fachärzt:innen Erwachsene | 19 | 19 | 0% |  | | 14,5 | 15,8 | 9% |
|  | Psychotherapeut:innen Erwachsene | 22 | 50 | 127% |  | | 22,0 | 35,5 | 61% |
|  | Psychotherapeut:innen KJP | 5 | 15 | 200% |  | | 5,0 | 13,0 | 160% |
| Landkreis Leipzig | Fachärzt:innen KJP |  | 2 |  |  | |  | 2,0 |  |
|  | Fachärzt:innen Erwachsene | 18 | 22 | 22% |  | | 11,0 | 16,8 | 52% |
|  | Psychotherapeut:innen Erwachsene | 26 | 55 | 112% |  | | 25,5 | 35,0 | 37% |
|  | Psychotherapeut:innen KJP | 6 | 16 | 167% |  | | 5,0 | 12,0 | 140% |
| Leipzig | Fachärzt:innen KJP | 13 | 13 | 0% |  | | 10,8 | 9,9 | -9% |
|  | Fachärzt:innen Erwachsene | 72 | 80 | 11% |  | | 58,7 | 61,5 | 5% |
|  | Psychotherapeut:innen Erwachsene | 174 | 264 | 52% |  | | 167,6 | 182,4 | 9% |
|  | Psychotherapeut:innen KJP | 47 | 61 | 30% |  | | 36,0 | 39,7 | 10% |
| Meißen | Fachärzt:innen KJP | 2 | 3 | 50% |  | | 1,5 | 1,8 | 20% |
|  | Fachärzt:innen Erwachsene | 13 | 24 | 85% |  | | 11,0 | 17,3 | 57% |
|  | Psychotherapeut:innen Erwachsene | 25 | 62 | 148% |  | | 23,2 | 38,9 | 68% |
|  | Psychotherapeut:innen KJP | 8 | 17 | 113% |  | | 3,0 | 9,0 | 200% |
| Mittelsachsen | Fachärzt:innen KJP | 1 | 1 | 0% |  | | 0,5 | 0,5 | 0% |
|  | Fachärzt:innen Erwachsene | 18 | 23 | 28% |  | | 14,5 | 18,0 | 24% |
|  | Psychotherapeut:innen Erwachsene | 43 | 64 | 49% |  | | 38,0 | 47,5 | 25% |
|  | Psychotherapeut:innen KJP | 11 | 18 | 64% |  | | 8,5 | 12,0 | 41% |
| Nordsachsen | Fachärzt:innen KJP |  | 1 |  |  | |  | 1,0 |  |
|  | Fachärzt:innen Erwachsene | 13 | 17 | 31% |  | | 9,0 | 11,5 | 28% |
|  | Psychotherapeut:innen Erwachsene | 12 | 48 | 300% |  | | 12,0 | 33,3 | 177% |
|  | Psychotherapeut:innen KJP | 6 | 14 | 133% |  | | 4,0 | 8,0 | 100% |
| Sächsische Schweiz-Osterzgebirge | Fachärzt:innen KJP | 1 | 2 | 100% |  | | 0,5 | 1,8 | 250% |
|  | Fachärzt:innen Erwachsene | 13 | 20 | 54% |  | | 10,5 | 17,0 | 62% |
|  | Psychotherapeut:innen Erwachsene | 33 | 57 | 73% |  | | 31,5 | 38,0 | 21% |
|  | Psychotherapeut:innen KJP | 8 | 17 | 113% |  | | 5,0 | 8,0 | 60% |
| Vogtlandkreis | Fachärzt:innen KJP | 3 | 2 | -33% |  | | 2,5 | 2,0 | -20% |
|  | Fachärzt:innen Erwachsene | 15 | 14 | -7% |  | | 13,0 | 13,0 | 0% |
|  | Psychotherapeut:innen Erwachsene | 31 | 41 | 32% |  | | 29,7 | 34,0 | 14% |
|  | Psychotherapeut:innen KJP | 7 | 12 | 71% |  | | 7,0 | 8,5 | 21% |
| Zwickau | Fachärzt:innen KJP | 3 | 2 | -33% |  | | 3,0 | 2,0 | -33% |
|  | Fachärzt:innen Erwachsene | 28 | 24 | -14% |  | | 22,0 | 20,0 | -9% |
|  | Psychotherapeut:innen Erwachsene | 63 | 74 | 17% |  | | 56,3 | 56,3 | 0% |
|  | Psychotherapeut:innen KJP | 16 | 25 | 56% |  | | 10,0 | 15,0 | 50% |
|  |  |  |  |  |  | |  |  |  |
| Sachsen Gesamt | Fachärzt:innen KJP | 36 | 48 | 33% |  | | 26 | 37 | 41% |
|  | Fachärzt:innen Erwachsene | 358 | 416 | 16% |  | | 288 | 321 | 12% |
|  | Psychotherapeut:innen Erwachsene | 761 | 1224 | 61% |  | | 706 | 827 | 20% |
|  | Psychotherapeut:innen KJP | 207 | 328 | 58% |  | | 152 | 212 | 39% |

*Abbildung A1 – Entwicklung der Anteile von Vertragsärzten mit kleiner gleich 50% bzw. mehr als 50% des vollen Versorgungsauftrages von 2014 bis 2023*


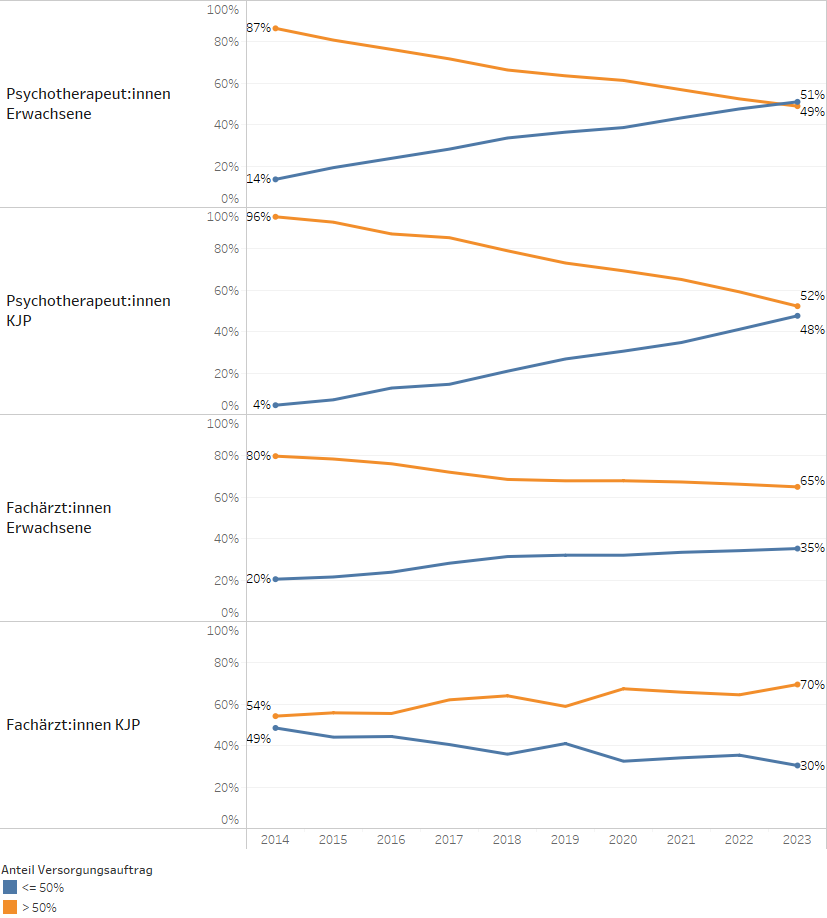

Supplement: Supplementary file 1 — Anlage [file 115_2025_1805_MOESM1_ESM.docx]
